# Microarray analysis of transcriptional responses to salt and drought stress in *Arabidopsis thaliana*

**DOI:** 10.1016/j.heliyon.2019.e02614

**Published:** 2019-11-29

**Authors:** Razieh Ghorbani, Abbas Alemzadeh, Hooman Razi

**Affiliations:** Department of Crop Production and Plant Breeding, School of Agriculture, Shiraz University, Shiraz, Iran

**Keywords:** Agriculture, Plant biology, Ecology, Genetics, Agronomy, Horticulture, *Lagenaria siceraria*, Cucurbits, Harvest time, Vivipary, Seed maturation, Yield, *Arabidopsis*, Meta-analysis, Transcriptomics, Transcription factors

## Abstract

Microarray expression profile analysis is a useful approach to increase our knowledge about genes involved in regulatory networks and signal transduction pathways related to abiotic stress tolerance. Salt and drought, as two important abiotic stresses, adversely affect plant productivity in the world every year. To understand stress response mechanisms and identify genes and proteins which play critical roles in these mechanisms, the study of individual genes and proteins cannot be considered as an effective approach. On the other hand, the availability of new global data provides us an effective way to shed some light on the central role of molecules involved in stress response mechanisms in the plant. A meta-analysis of salt and drought stress responses was carried out using 38 samples of different experiments from leaves and roots of *Arabidopsis* plants exposed to drought and salt stresses. We figured out the number of differentially expressed genes (DEGs) was higher in roots under both stresses. Also, we found that the number of common DEGs under both stresses was more in roots and also the number of common DEGs in both tissues under salt stress was more than drought stress. The highest percent of DEGs was related to cell and cell part (about 87%). Around 9% and 7% of DEGs in roots and leaves encoded transcription factors, respectively. Network analysis revealed that three transcription factor families HSF, AP2/ERF and C2H2, may have critical roles in salt and drought stress response mechanisms in *Arabidopsis* ​and some proteins like STZ may be introduced as a new candidate gene for enhancing salt and drought tolerance in crop plants.

## 1 Introduction

Plants unlike animals are not able to move and escape from undesirable condition and stresses. Abiotic stresses such as drought, salt, extreme temperature, heavy metals and nutrient deficiency have undesirable effects on plants [1]. These stresses cause decrease in growth and productivity of plants. Among these stresses, drought and salt are two major environmental factors which have detrimental effects on growth, yield and crop production. There is severe water shortage in the world and the majority of these water resources are exposed to salt. Hence, it is necessary to improve water use efficiency and salt tolerance of crops through plant breeding projects using new tools like biotechnology [2].

Plant have responded to salt and drought stresses at the molecular and cellular levels and also at the physiological and biochemical levels [3]. There are different mechanisms evolved in plants to receive and transmit signals from the environment to cells which leads to response to different stresses [4]. Molecular biology techniques together with bioinformatic studies have provided a powerful tool to identify a number of genes and proteins involving in different pathways of stress response mechanisms (SRMs) [5, 6]. Molecular response of plants to environmental stresses may actually involve a complex interaction between different molecules related to various pathways. Meta-analysis is the statistical approach which combine results from different studies to clarify simultaneously expression of thousands of genes and could be used to detect key genes in response to different stresses [7]. Recently, meta-analysis methods enable scientists to simultaneously study different molecules such as transcripts and proteins under various conditions [8].

The stress-inducible genes that their products involved in the stress may be classified into two groups: the first group including those that protect the cells against stress; and the second group including those that have crucial roles in gene expression under stress conditions [9].

Determining common proteins and their encoding genes between different stresses will allow us to shed some light on the SRMs in plants. Recent years have witnessed an upsurge in the number of functional molecular studies reporting crosstalk in different pathways involved in stress response mechanisms. There are some meta-analysis data from different plants revealing important genes which have hub roles in different abiotic stresses [6, 10]. Among the plants, *Arabidopsis* ​as a model plant has been subjected to a number of meta-analysis studies which leads to finding differentially expressed genes (DEGs) between stress and non-stress conditions [8, 11, 12, 13]. Detection of DEGs is the first step in understanding of SRMs in plants [13]. Recent advances in biotechnology and development of new technologies caused to facilitate the detection of new genes and determine their functions. Genomics, transcriptomics, proteomics and metabolomics may be used to identify important genes related to abiotic stress response and determine their roles in different pathways involved in SRMs [14]. One of the most important molecules which play crucial roles in these mechanisms are transcription factors [6]. There are some reports showed the roles of some transcription factors family in regulation of stress-inducible genes under stress conditions [6, 9, 15].

Gene ontology (GO) enrichment has been used as a powerful tool in many studies to determine the molecular activity of a gene (molecular function), determine the physiological role of a gene product and coordination with other genes (biological process) and determine the location of gene product in the cell (cellular component). Also, GO is usually used to annotate the function of genes and their proteins from various species especially model organisms such as *Arabidopsis*.

The aim of this study was to identify genes involved in drought and salt stress responses in *Arabidopsis* ​using microarray gene expression data by meta-analysis approach. In addition, we aimed to detect DEGs between stress and non-stress conditions. Moreover, in this research it was tried to figure out most important transcription factors involved in theses stresses that affect the expression of various genes through their down- or up-regulation. In this paper, the functions of identified DEGs and transcription factors were discussed and using network and GO analysis, the complexity of SRMs in *Arabidopsis* ​was explained.

## 2 Materials and methods

### 2.1 Gene expression data collection

The raw microarray expression data was downloaded from ArrayExpress database (https://ebi.ac.uk/arrayexpress/) to identify drought and salt stress-responsive genes in *Arabidopsis*. The data originated from Affymetrix microarray platform. These datasets obtained from 6 independent experiments with 38 samples from leaves and roots of plants exposed to drought and salt stresses. These data were obtained from series IDs E-MEXP-1863, E-GEOD-48474 and E-GEOD-40061 (response to drought stress), and E-MEXP-2858, E-GEOD-53308 and E-GEOD-71001 (response to salt stress). All experiments were performed under 22–25 °C, 65–90% RH (relative humidity) and 16/8 h light/dark cycle in the growth chamber. Wild-type Columbia-0 was used in all experiments. In the E-MEXP-1863 experiment, 3-week-old plants were subjected to drought stress by withholding water for 5 days until plant samples reached of 52.5 ± 7.5% relative water content (RWC). In the E-GEOD-48474 experiment, drought stress was applied to 2-week-old WT plants were by withholding water for 21 days. Leaf sample were collected for transcriptome analysis in mentioned experiments. In the E-GEOD-40061 experiment, root sample were collected from 3-week-old plants which were exposure under drought stress using withholding water for 14 days (soil moisture below 30%). In the E-MEXP-2858 experiment, leaf sample were collected from one-month plants which treated with 200mM NaCl for seven days for salt treatment. In the E-GEOD-53308 experiment, leaf sample were collected from one-month plants which treated with 150 mM NaCl for four days for salt treatment. In the E-GEOD-71001 experiment, root sample were collected from 6-week-old plants which treated with 150 mM NaCl for 6 h.

### 2.2 Detection of DEGs

The data were retrieved from ArrayExpress database and then analyzed to obtain DEGs involved in responses to drought and salt stresses. The downloaded raw data in format CEL data were analyzed by FlexArray software version 1.6.3. Data were normalized by Robust Multi-array Average (RMA) algorithm using R version 3.2.2. To identify DEGs, the processed microarray data were subjected to two-sample student's t-test. Up-regulated genes with significant *P*-value (false discovery rate (FDR) < 0.05) with fold change value greater than 1.5 selected as DEGs [6, 11]. Venn diagram of DEGs was created by Venny 2.0 (http://bioinfogp.cnb.csic.es/tools/venny/).

### 2.3 Detection of transcription factors

The selected DEGs were further compared with the transcription factors genes in the PlantTFDB server http://planttfdb.cbi.pku.edu.cn/index.php/ for identification of genes encoding transcription factors.

### 2.4 Gene ontology of DEGs

GO enrichment analysis of DEGs was performed using the AgriGO tool (http://bioinfo.cau.edu.cn/agriGO/) with default parameters such as Fisher statistical test method, Multi-test adjustment method of Yekutieli (FDR under dependency) with significant level of 0.05 and complete GO gene ontology type [16]. A Singular Enrichment Analysis (SEA) was performed using TAIR genome as reference and FDR less than <0.05.

### 2.5 Interaction networks between DEGs

Functional associations between DEGs during drought and salt stresses were figured out by STRING v.10 (Search Tool for the Retrieval of Interacting Genes/Proteins) database (https://string-db.org/). These associations were constructed based on co-expression data which is stored in the NCBI-GEO database, co-occurrence of the genes in the same organisms, text mining which is a list of significant protein interaction groups, extracted from the abstracts of scientific literature, significant protein interaction datasets from other experiments, individual gene fusion events per species, significant protein interaction groups gathered from curated databases and neighborhood which is runs of genes that occur repeatedly in close neighborhood in genomes. Confidence score for interactions was medium score (above 0.4). The gene network obtained from STRING v.10 was imported into Cytoscape version 3.6.0 for further analysis and display.

## 3 Results and discussion

### 3.1 Identification of DEGs in *Arabidopsis thaliana* under stress conditions

**Fig. 1:**
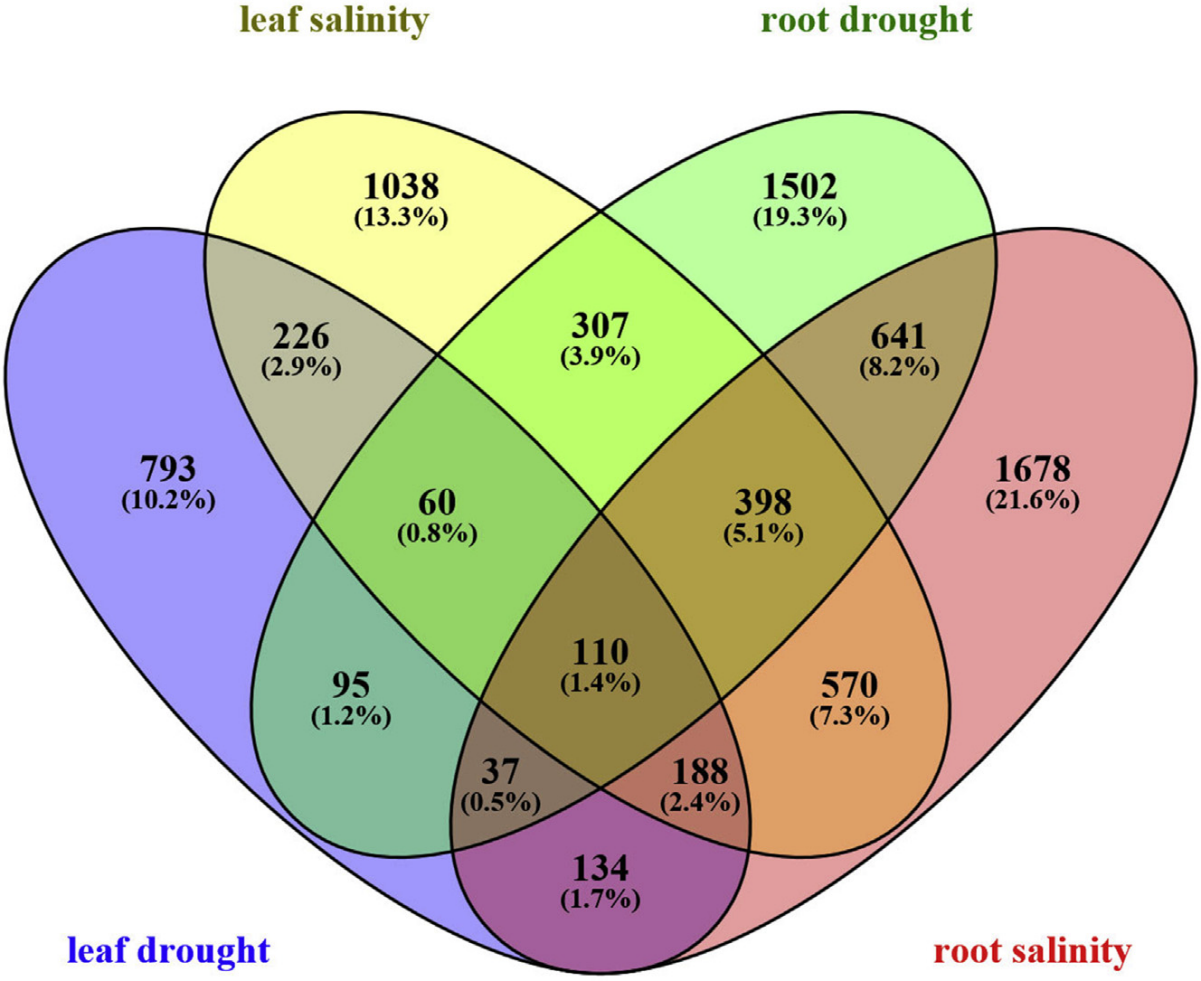
Venn diagram of differentially expressed genes in response to drought and salt stresses in leaves and roots of *Arabidopsis thaliana* which created by Venny 2.0.

This study was carried out to achieve more information about the changes in gene expression in response to drought and salt stresses in leaves and roots of *Arabidopsis*. Through comprehensive comparison analysis, we identified a set of 4540 up-regulated DEGs in the leaf tissue which 1643 (36%) and 2897 (64%) of them up-regulated in response to drought and salt stress, respectively, whereas in the root tissue 6906 DEGs were up-regulated that 3150 (46%) and 3756 (54%) of them up-regulated in response to drought and salt stress, respectively (supplementary 1). The number of DEGs which up-regulated in roots was 1.5-fold higher than leaves that under drought stress was 1.9-fold, whereas under salt stress was less than 1.3-fold. A four-way Venn diagram was used to represent co-occurrence of DEGs under salt and drought stresses in root and leaf tissues. The diagram represented a list of 584 (13%) DEGs was up-regulated under salt and drought stresses in leaf tissue, but 1186 (17%) DEGs were common between drought and salt stresses in roots (Fig. 1). In the other words, the number of DEGs involved in both stresses in roots was 2-fold higher than leaves. Also, the common DEGs between roots and leaves under drought and salt stresses were determined using the Venn diagram. Under salt stress, 1266 (11%) DEGs were common between root and leaf tissues, but the number of common DEGs under drought stress was only 302 (2.6%), that means the number of common DEGs in both tissues under salt stress was 4.2-fold higher than drought stress (Fig. 1). It can be concluded that the number of common pathways between roots and leaves induced by salt stress is more than those of induced by drought stress.

As mentioned above, the number of DEGs was up-regulated under stress conditions in roots was higher than leaves that point to the important role of roots in abiotic SRMs in *Arabidopsis*. In some plants like cotton it has been reported that the number of DEGs in leaves was higher than roots under salt stress [17], but there are some reports in other plants which showed the number of up-regulated DEGs in roots were higher than leaves under drought stress [18]. It indicates that different species may use different tolerance mechanisms in response to different stresses. The root is the first tissue to sense stress conditions and it may be explained why, in some plants like *Arabidopsis*, the number of genes involved in the stress in root tissue is higher than other tissues, but in some plants like cotton, probably, leaves have a central role in the control of water loss and hence, more genes involved in the stress in this tissue [19]. According to their stress response mechanisms, plants may be divided into classes: (i) plants that root has a central role and (ii) plants that the leaf has a central role.

The expression changes of DEGs was from 1.5 to 137-fold respect to the control (supplementary 1). The percent of DEGs which up-regulated more than 10-fold in response to drought stress in the root tissue (3.1%) was more than the leaf tissue (1.6%), but it was reverse in the case of salt stress, in roots, 1.6% of DEGs up-regulated more than 10-fold, while it was 2% for leaves (supplementary 1). These results indicate that the genes in leaves and roots have different expression patterns in response to salt and drought stresses.

### 3.2 Gene ontology

Gene ontology (GO) enrichment analysis was conducted to detect the function of up-regulated DEGs in response to drought and salt stresses in root and leaf tissues in *Arabidopsis*. A total of 1617 and 2854 DEGs respectively in drought and salt stresses in leaves and 3026 and 3628 DEGs respectively in drought and salt stresses in roots were analyzed for GO analysis. Based on GO analysis, up-regulated DEGs were categorized into 3 groups of biological process (BP), molecular function (MF) and cellular component (CC).

**Fig. 2:**
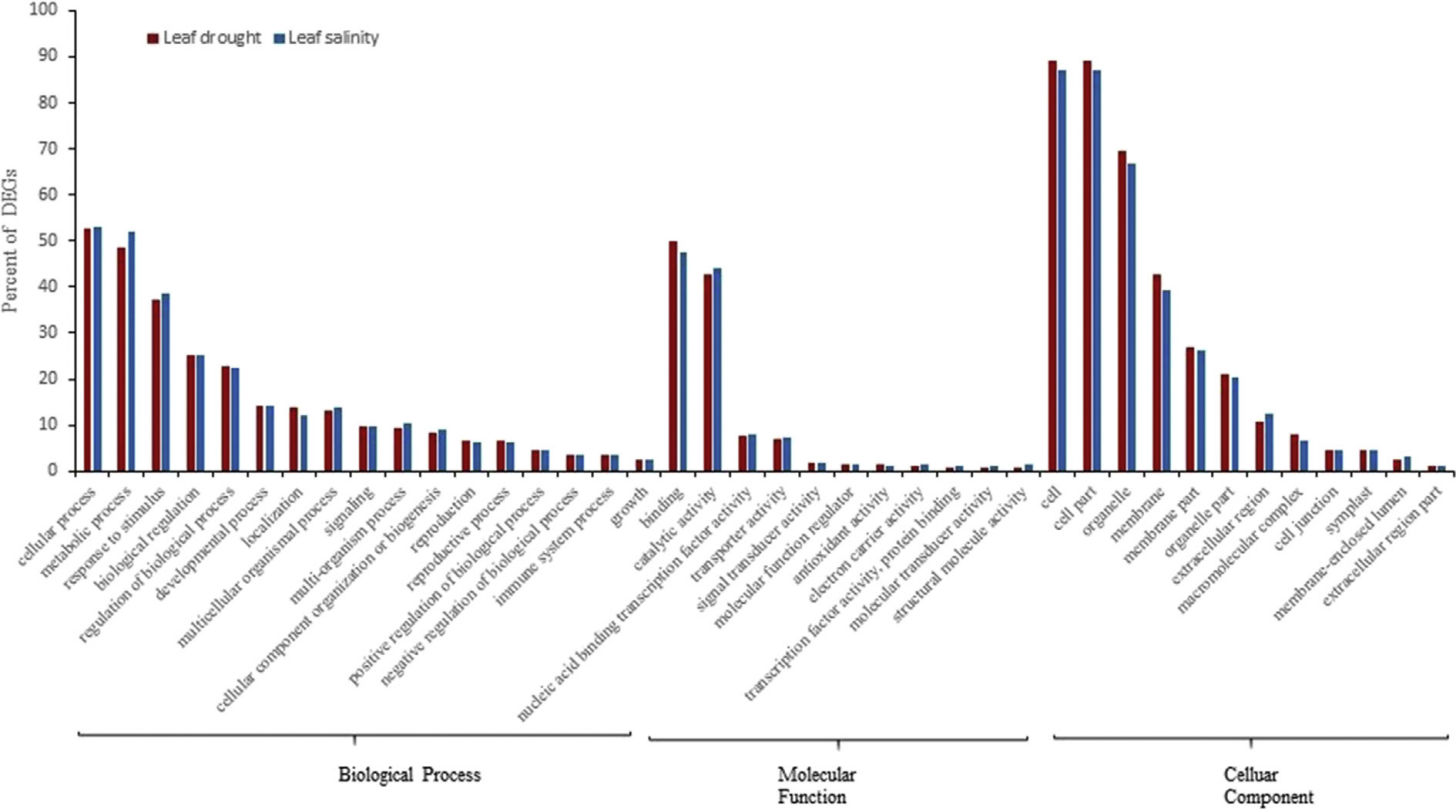
Gene ontology analysis in leaves. Frequency of most representative biological process terms in *Arabidopsis thaliana* under salt and drought stresses. Gene ontology analysis was made in the AgriGO platform (FDR = 5%). More details in Supplementary 2.

**Fig. 3:**
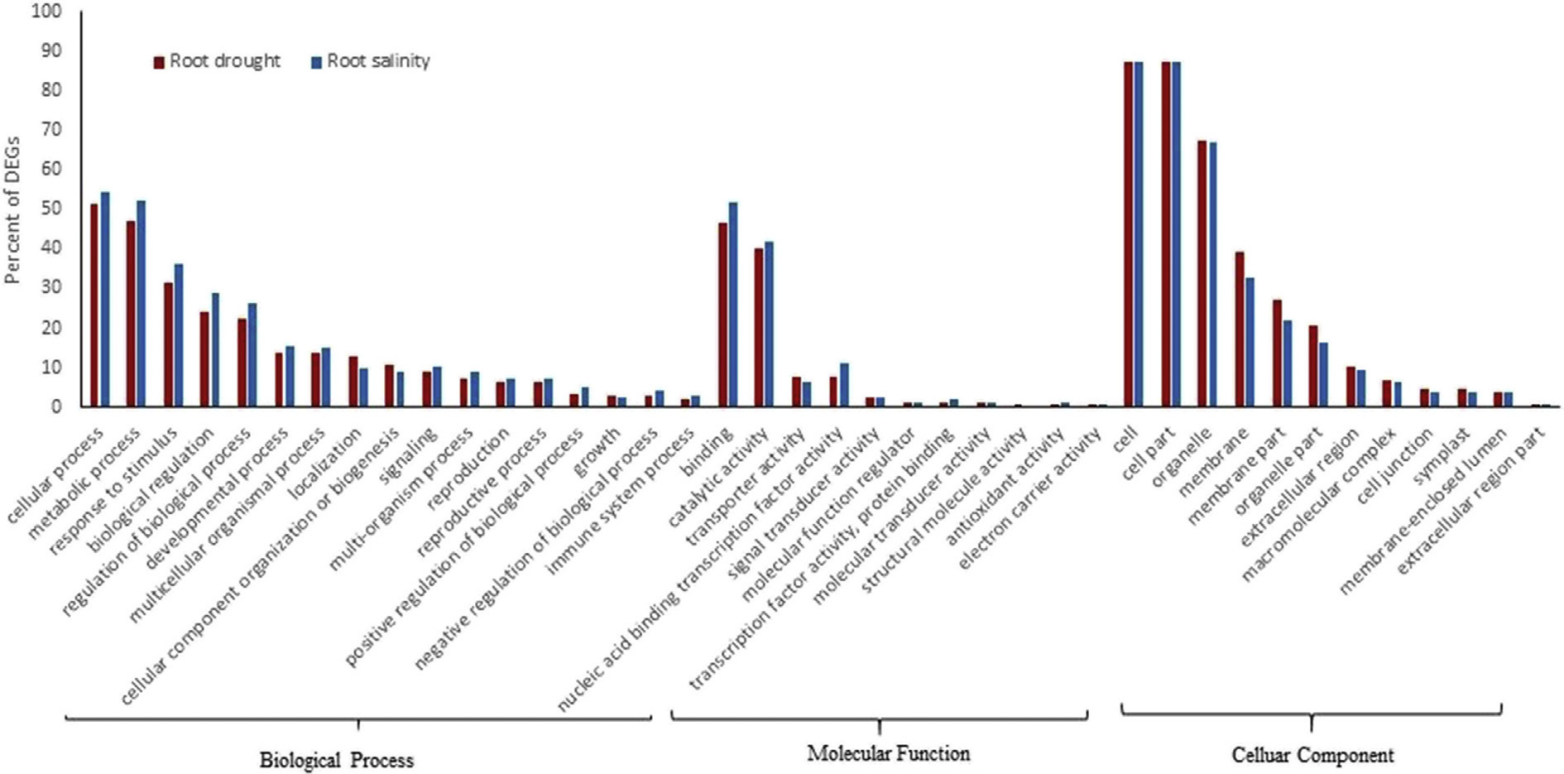
Gene ontology analysis in roots. Frequency of most representative biological process terms in *Arabidopsis thaliana* under salt and drought stresses. Gene ontology analysis made in the AgriGO platform (FDR = 5%). More details in Supplementary 2.

The aforementioned groups, BP, MF and CC consisted of 17, 11 and 12 categories, respectively (Figs. 2 and 3). In both tissues, in response to both stresses, for CC most enriched GO terms were cell (about 87%), cell part (about 87%) and organelle (about 65%), whereas the two highly most enriched ones for MF were binding (49%) and catalytic activity (42%) (Figs. 2 and 3). Furthermore, for BP the highest frequency of DEGs function was belonged to cellular process (53%), metabolic process (50%) and response to stimulus (36%), respectively (Figs. 2 and 3), which agreement with results of other researches about microarray meta-analysis in plants in response to biotic and abiotic stresses [6, 20].

In BP, the significant DEGs in cellular process (GO:0009987) were assigned to cellular response to stimulus, regulation of cellular process, multi-organism cellular process, cellular metabolic process, single-organism cellular process and positive regulation of cellular process (supplementary 2). For the second group in BP, metabolic process (GO:0008152), the significant ones were assigned to GO terms such as single-organism metabolic process, cellular metabolic process, organic substance metabolic process and catabolic process.

In BP group, for signaling (GO:0023052) and response to stimulus (GO:0050896), the number of DEGs in each tissue in salt stress was higher than drought stress. In signaling group, 9.5 and 9.7 percent of DEGs in the leaf and 9.1 and 10.3 percent of DEGs in the root were up-regulated in response to drought and salt stresses, respectively (Figs. 2 and 3). Gene ontology of up-regulated DEGs showed that signaling category included DEGs involvement in hormone-activated signaling pathways, signal transduction, MAP kinase kinase activity, ATPase activity, calcium-dependent protein serine/threonine kinase activity and receptor signaling protein serine/threonine kinase activity. Phytohormones such as abscisic acid, auxin, cytokinin and gibberellic acid play important roles in different process of growth and development and also response to biotic and abiotic stresses in plants [21, 22]. In Chai *et al* (2019) study, Expression of At*NCED3* (nine-cis-epoxycarotenoid dioxygenase) which is an important gene in ABA biosynthesis, increased about 4.6-fold in *Arabidopsis* ​plants after salt stress. Also, in our study salt stress increased expression of this gene about 1.8-fold in leaf tissue of *Arabidopsis* ​plant (supplementary 1).

Another major part of up-regulated DEGs in BP group, was belonged to response to stimulus category. Based on GO results, 37 and 38 percent of DEGs in leaves and 31 and 36 percent of DEGs in roots were up-regulated in response to drought and salt stresses, respectively (Figs. 2 and 3). Components of this group were up-regulated in response to abiotic stimulus, such as temperature, salt and drought stresses, response to endogenous stimulus such as phytohormones.

Components of MF group have important roles in response to abiotic stresses. In this category, catalytic activity and binding groups have more up-regulated DEGs in both tissues. Majority of up-regulated DEGs (>46%) was belonged to binding category (GO:0005488) and more than 40% was belonged to catalytic activity category (GO:0003824) in each tissue (Figs. 2 and 3). The results were coincidence with that obtained by Gao *et al* (2008) in response to drought stress in chickpea [23].

Some significant GO terms of binding group were ion binding, lipid binding, cofactor binding and protein binding and significant GO terms of catalytic activity group were oxidoreductase activity and hydrolase activity. Catalytic activity role in response to abiotic stresses especially osmotic stress was demonstrated [24]. Based on previous studies, antioxidant activity plays an important role in abiotic and even biotic stresses tolerance, but despite its important role in plant defense against osmotic stress, amount of up-regulated DEGs in this group was low in each tissue and in each stress, so that amount of this group was 1.4 and 0.94 percent in leaf and 0.85 and 1.1 percent in root respectively under drought and salt stresses. This kind of results has also been reported in earlier studies [25]. Genes encoded antioxidant activity are one of the most important genes in response to abiotic stresses. In plants during of abiotic and biotic stresses, reactive oxygen species (ROS) generated which severe damage to whole cells and organisms. Antioxidant enzymes such as superoxide dismutase (SOD), catalase (CAT), ascorbate peroxidase (APX), glutathione peroxidase (GPX), and glutathione reductase (GR) scavenge ROS components in plants [25, 26]. Kanesaki *et al* (2002) identified which salt stress caused to increase the expression of superoxide dismutase in *Synechocystis*. In our study, this gene up-regulated in response to drought and salt stress in leaf tissue[27]. Furthermore, Ghaffari *et al* (2013) reported an increase of ascorbate peroxidase 2-like protein and Cu/Zn superoxide dismutase in a tolerant line of sunflower roots in response to drought stress [28]. In our study, the expression of ascorbate peroxidase gene increased in root tissue in response to drought and salt stress. Based on results of Li *et al* (2012), osmotic stress using polyethylene glycol (PEG), increased the expression of monodehydroascorbate reductase (MDHAR) in leaves of wild type tomato. MDHAR play important role in plant antioxidant system by regeneration of ascorbate. In our study, the expression of this gene also up-regulated in leaf tissue in response to salt stress.

Transcription factors play important roles in plant responses to stresses via regulation of downstream genes by binding to *cis*-acting elements in the promoter region of target genes [29]. The percent of these proteins were low in our study as previously reported by Khojasteh *et al* (2018).

The results of GO analysis showed a similarity pattern of DEGs function for leaf and root tissues in response to drought and salt stresses but differences were in specific DEGs and number of significant DEGs in each group between two stresses in each tissue (supplementary 2). In roots, approximately in most categories of BP and MF groups, amount of up-regulated DEGs under salt stress were higher than drought stress, but in the CC group, the amount of up-regulated DEGs was approximately equal under both stresses except for membrane, membrane part and organelle part categories which amount of up-regulated DEGs was higher in drought stress. Whereas in leaf tissue, the amount of up-regulated DEGs in different categories of gene ontology was not so much different between two stresses. These results may indicate that roots respect to salt stress is more sensitive than drought stress.

### 3.3 Identification of DEGs encoding transcription factors in *Arabidopsis* in response to drought and salt stresses

Transcription factors (TFs) play important roles in plant tolerance to environmental stresses. These proteins as *trans*-acting elements bind to *cis* elements in promoter region of various genes involved in different stresses and as a result cause to activate or repress the expression of target genes and subsequently increase the plants tolerance [30]. With regard to the important role of transcription factors in plant response to different stresses, we further identified and compared the expression profiles of these proteins in root and leaf tissues of *Arabidopsis* ​under drought and salt stresses.

**Fig. 4:**
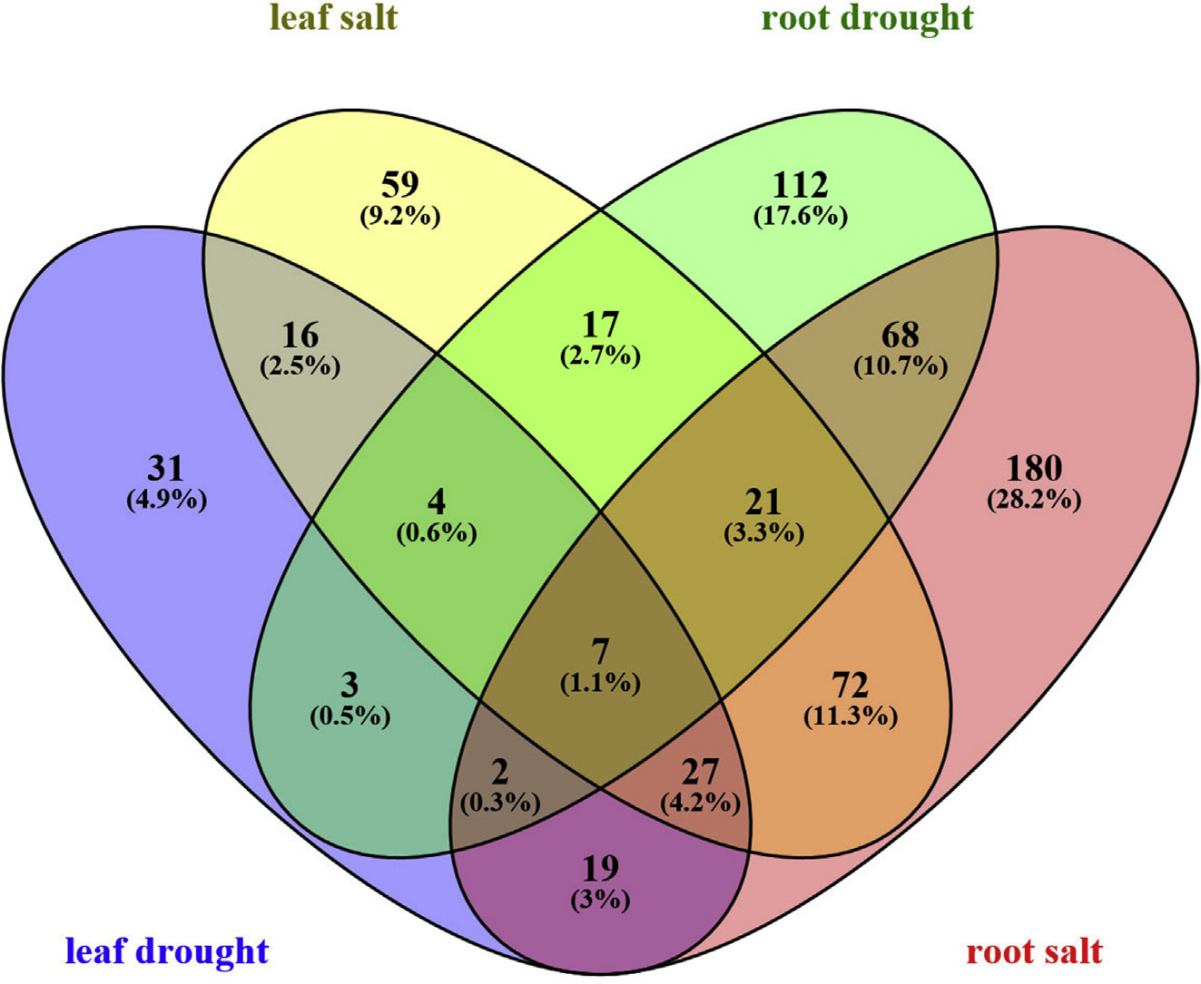
Venn diagram of differentially expressed genes encoding transcription factors in response to drought and salt stresses in leaves and roots of *Arabidopsis thaliana* which created by Venny 2.0.

**Fig. 5:**
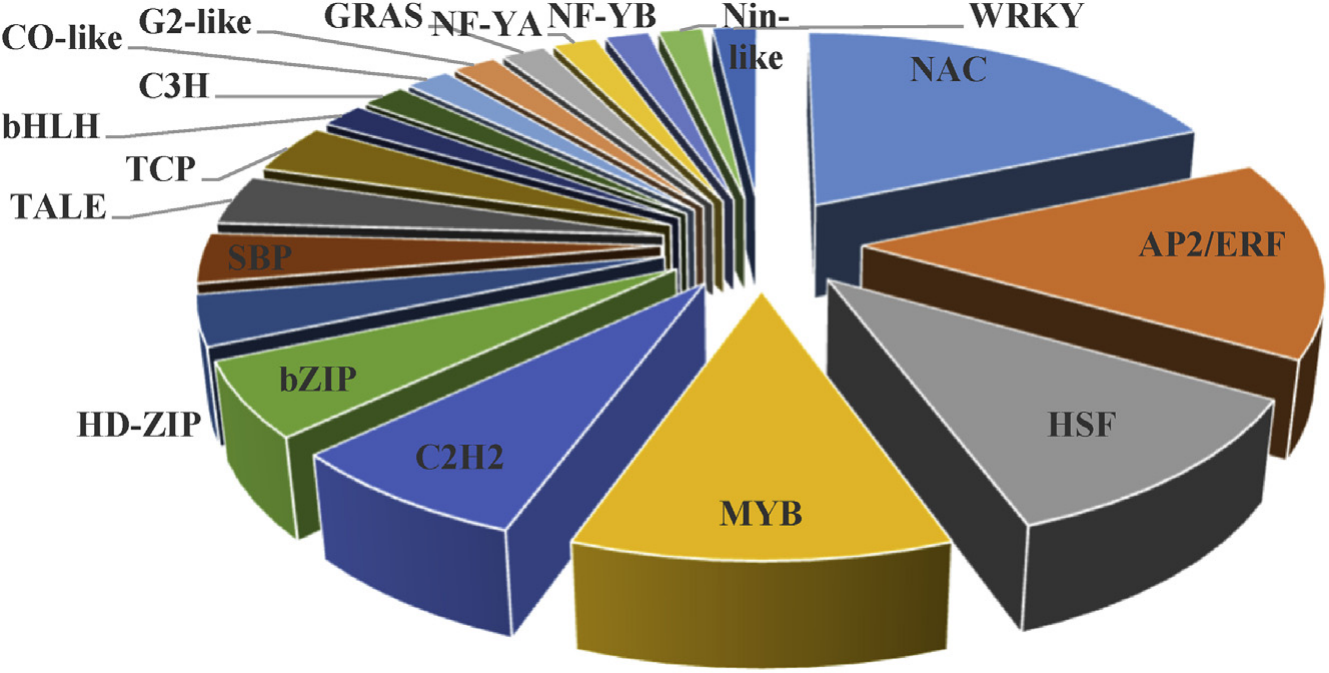
Common transcription factors in leaves of *Arabidopsis thaliana* which up-regulated in response to drought and salt stresses.

**Fig. 6:**
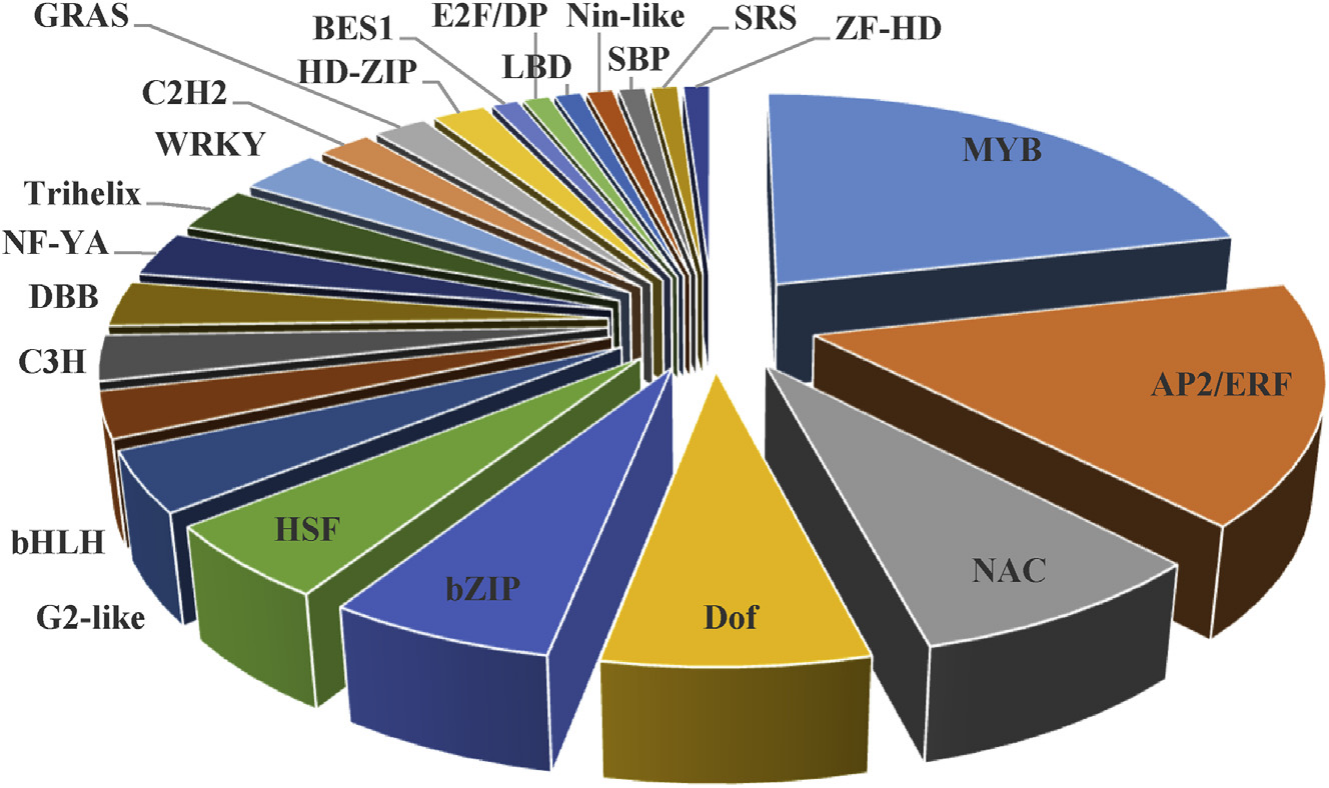
Common transcription factors in roots of *Arabidopsis thaliana* which up-regulated in response to drought and salt stresses.

Based on the results, 109 (about 6.6%) and 223 (about 7.7%) genes in leaves and 234 (about 7.4%) and 396 (about 10.5%) genes in roots respectively in response to drought and salt stress were identified as TFs (Fig. 4). Identified TFs were classified in 28 and 34 groups in leaves, and 37 and 40 groups in roots in response to drought and salt stress, respectively (supplementary 3). In each tissue under both stresses, the greatest TF families were belonged to AP2/ERF, MYB and NAC families. Moreover, other important TF families such as bZIP, HSF, C2H2 and WRKY were also detected. In addition, 54 and 98 TFs respectively in leaf and root tissues were identified as common TFs between drought and salt stresses (supplementary 4). Common TFs in leaves were divided into 19 groups. The greatest number of these TFs was belonged to NAC family followed by AP2/ERF, HSF, MYB, C2H2 and bZIP accounting for 68% of the total TFs (Fig. 5). Furthermore, common TFs in roots were divided into 23 groups which the greatest number of them was belonged to MYB family followed by AP2/ERF, NAC, DOF, bZIP and HSF accounting for 64% of the total TFs (Fig. 6).

AP2/ERF family is one of the most important transcription factors family in plants which plays important roles in response to biotic and abiotic stresses [31, 32, 33]. In the present study, *CRF7* (AT1G22985, CYTOKININ RESPONSE FACTOR 7), a member of ERF family, was up-regulated in response to drought and salt stresses in leaves whereas its expression in roots increased only under salt stress (supplementary 3). The analysis of *Arabidopsis* ​microarray data previously showed that the expression of CRF genes changed in response to abiotic stresses [34]. Overexpression of *HARDY* (AT2g36450) gene, encoding a member of this family, improved drought and salt tolerance in transgenic *Trifolium alexandrinum* L [35]. Our microarray analysis results also showed that the expression of this gene up-regulated in roots in response to drought stress (supplementary 3). *RAP2.1* (AT1G46768) which up-regulated in response to both stresses in leaves (supplementary 3), encodes a member of the DREB subfamily. It has previously shown that drought and cold stresses induced expression of *RAP2.1* gene through ABA-independent pathway [36]. The expression of *RAP2.1* gene is negatively regulated by cold and drought stresses in *Arabidopsis*. Moreover, *RAP2.6* (AT1G43160) and *DREB19* (AT2G38340) which encodes a member of ERF and DREB subfamilies, respectively, up-regulated in roots in response to drought and salt stresses, whereas up-regulated only in response to salt stress in leaves (supplementary 3). In addition, it has been reported that the expression of these genes significantly up-regulated under salt stress in transgenic plants [36]. It has been previously reported that the expression of *DREB2A* (AT5G05410), a gene which encodes a member of DREB subfamily, increased in response to salt, drought and heat stresses [32, 37]. One of the earliest responses to drought stress is activation of *ERF* genes such as *ERF8* (AT1G53170) which results in the induction of ethylene biosynthesis [38]. The expression of both of these genes, *DREB2A* and *FRF8*, was up-regulated in response to salt and drought stress in leaves, whereas up-regulated only under salt stress in roots (supplementary 3). *Arabidopsis* transgenic plants contain *TaERF1* showed tolerance to different stresses such as drought, salt and low-temperature stresses [39]. It has been previously shown that *ERF104* (AT5G61600) and *ERF105* (AT5G51190) which in this study up-regulated in leaves under both stresses (supplementary 3), play a critical role in response to high light and freezing stresses [40].

Another important transcription factor family in plants is NAC family. There are some reports that showed the members of this family has key roles in response to biotic and abiotic stresses [41]. In *Arabidopsis*, 105 redundant putative NAC genes were identified [42]. Our results showed that *NAC019* (AT1G52890), *NAC02* (AT5G04410) and *NAC072* (AT4G27410, *RD26*) were up-regulated in leaves in response to both stresses, while their expression increased in roots only under salt stress, whereas *NAC01* (AT1G01010) and *NAC032* (AT1G77450) were up-regulated in roots in response to both stresses and *NAC032* was up-regulated only in response to salt stress in leaves. *NAC047* (AT3G04070) was up-regulated in both tissues in response to both stresses (supplementary 3). It has been shown that the Introduction of *NAC019* in *Arabidopsis* ​plants increased the tolerance to drought, salt, and low-temperature stresses in transgenic plants [43]. Different studies showed that other members of this family such as *OsNAC1*, *OsNAC2*, *OsNAC10* and *AtNAC2* have important roles in various abiotic stresses [44, 45]. Furthermore, it has been also demonstrated the role of some members of this family in ABA-dependent stress-signaling pathway [44, 46].

bZIP family is another large family of transcription factor in plants and 75 members of this family was recognized in *Arabidopsis* ​[47]. Microarray analysis of *Arabidopsis* in response to drought stress showed that the expression of *bZIP44* (AT1G75390) increased significantly in shoot tissue [48]. Our results indicated that its expression increased in response to both stresses in roots, whereas in leaves, up-regulated only in response to drought stress (supplementary 3). Moreover, in this study, other members of bZIP family such as *AtbZIP50/TGA7* (AT1G77920), *AtbZIP55/GBF3* (AT2G46270) were detected in response to drought and salt stresses in leaves, whereas *AtbZIP56/HY5* (AT5G11260) up-regulated in response to both stresses only in roots and in leaves up-regulated only under salt stress (supplementary 3).

MYB and WRKY are two families of transcription factors involved in response to abiotic stresses in plants [48]. For example, it has been shown that the expression of *AtMYB108/BOS1* gene induced in response to oxidative stress, drought and salt stresses. *AtMYB108* also involved in the crosstalk between abiotic and biotic stresses [49]. *AtMYB65* (AT3G11440) is a member of this family which involves in GA signaling in growth and flowering processes [50] and here, up-regulated in response to both stresses in roots (supplementary 3). *AtMYB108* (AT3G06490) regulated filament elongation and anther dehiscence by JA and GA signaling pathways. *AtMYB108* is involved in the crosstalk between abiotic and biotic stress signaling. Furthermore, the expression of *AtMYB108/BOS1* was induced in response to oxidative stress, drought and salt stresses [49]. Our work also showed that the expression of this gene increased in roots in response to both stresses whereas, in leaves up-regulated only in response to salt stress (supplementary 3). The results of the study showed that the expression of AT5G60890 (At*MYB34*) up-regulated in root tissue in response to both stresses, whereas in leaves, its expression increased only in response to salt stress. Up-regulation of this gene was identified in *Arabidopsis* ​plant in Bhargava *et al* (2013) study by microarray and RNA-Seq analysis.

It has been shown that there are 74 members for WRKY family in *Arabidopsis* ​[51]. *AtWRKY25* (AT2G30250), a member of WRKY family, expresses under different abiotic stresses such as heat, drought, salt and oxidative stresses [52, 53, 54]. Here also, its expression increased in response to both stresses only in leaves but in roots, its expression increased just in response to salt stress (supplementary 3). In other species, it has also reported that the members of these families have important roles in abiotic stress responses. *OsWRKY23* (01g53260), as member of these family in rice, involved in the regulation of resistance to salt, ABA, H_2_O_2_, osmotic and dark stresses [55]. They showed that the overexpression of *OsWRKY23* in *Arabidopsis* ​caused to accelerate the leaf senescence in darkness.

Another important transcription factor family is HSF family. There are 21 genes which encodes the members of this family in *Arabidopsis* [56]. Our results indicated that some members of HSF family, *HSFA2*, *HSFB2B*, *HSFA4A*, *HSFA6A* and *HSFB2A* in leaves and *HSFA2*, *HSFB3*, *HSFA6B* and *HSFA3* in roots were up-regulated in response to both of drought and salt stresses (supplementary 3). These results are in accord with what others reported. Overexpression of *AtHSFA2* in *Arabidopsis*, increased the tolerance of plants against abiotic stresses [57]. These transcription factors are regulated by other proteins, for example, the transcription of *AtHSFA3* is regulated by *DREB2A* gene via binding to two DRE core elements of *DREB2A* in the *AtHSFA3* promoter region [58]. These TFs have important roles in abiotic stresses in other species, it has been shown that the expression of tomato *HSFA3* and wheat *HSF3* increased thermotolerance of *Arabidopsis* transgenic plants [59, 60]. The result of this study showed that the transcription factors family of AP2/ERF, MYB, NAC, bZIP, HSF and WRKY may play important roles in response to drought and salt stresses in *Arabidopsis*.

### 3.4 Gene network analysis

**Fig. 7:**
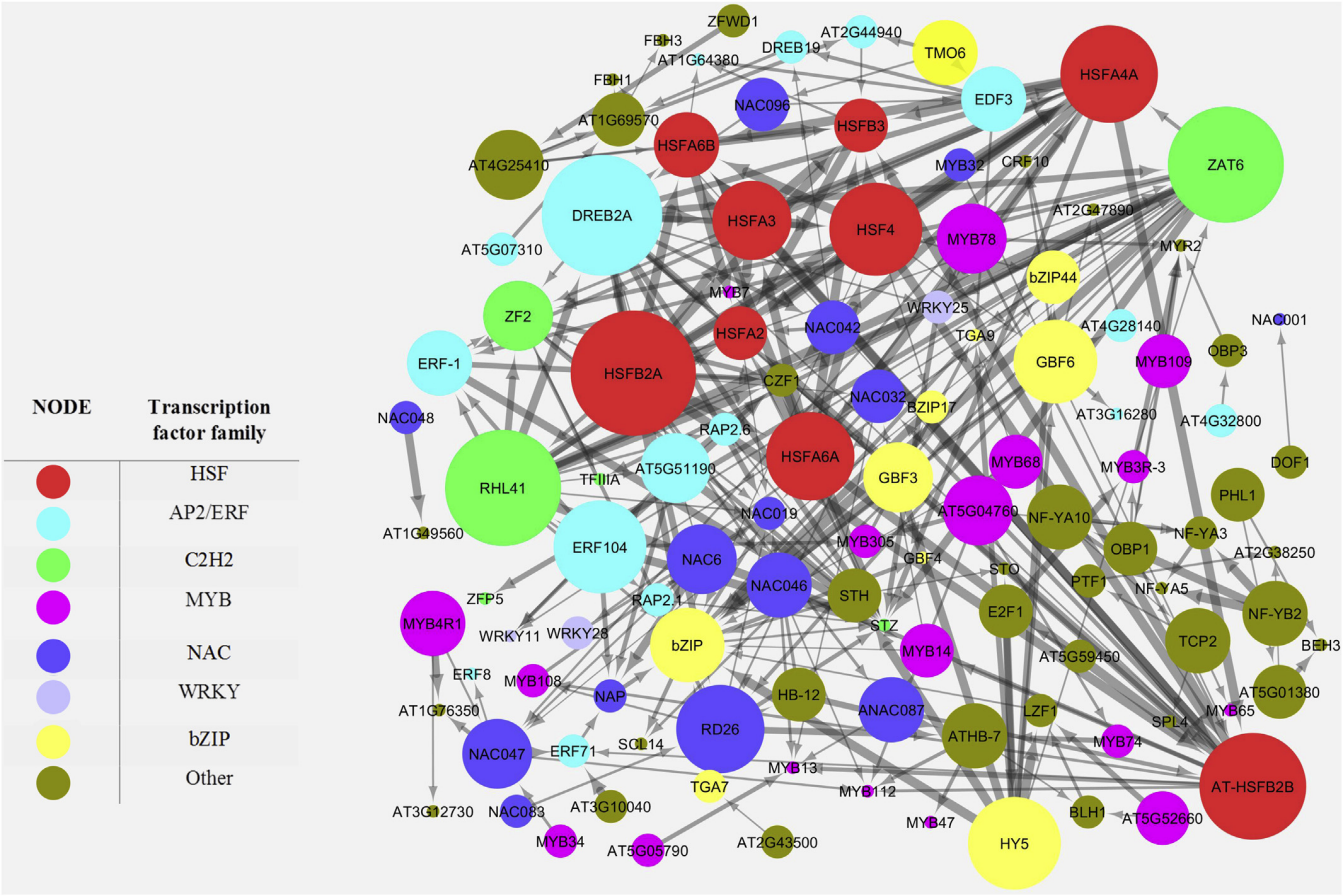
Network analysis of differentially expressed genes encoding transcription factors detected in salt and drought stresses. Network produced by STRING (version 10) with a confidence score >0.4, and visualized by Cytoscape 3.6.0. Node colors refer to the family of transcription factor. Node size corresponds to the number of outdegrees.

In this study, a network was generated between common TFs in leaves and roots in response to drought and salt stresses in *Arabidopsis*, using STRING 10.5. Only 113 of 152 TFs had interactions (supplementary 5) and so all the disconnected nodes in the network were removed. Between the TFs, seven of them, *HSFA2*, *HSF4*, *MYB47*, *MYB32*, *NAC047*, *NF-YA5* and AT1G76580 were common in both tissues and both stresses. Subsequently, the TFs which had interactions together, were subjected to Cytoscape v2.8.2 for more analysis. Based on the results of Cytoscape, a total of 550 interactions (edges) were detected between 113 TFs (nodes) (Fig. 7, supplementary 6). In this network, hub genes were identified based on their outdegree (the number of genes which was affected by the same gene) and those that had an outdegree equal or more than 10 considered as a hub gene which includes *HSFB2A* (belongs to HSF family), *DREB2A* (belongs to AP2/ERF family), *ZAT6* (belongs to C2H2 family), *RHL41* (belongs to C2H2 family), *HSFB2B* (belongs to HSF family) and *HSFA4A* (belongs to HSF family) (supplementary 6). These results indicated that the members of HSF, AP2/ERF and C2H2 families appear to play critical roles in salt and drought stress response mechanisms.

*HSFB2A* had the highest outdegree in the network, so that 16 genes, *RHL41*, *ZAT6*, *DREB2A*, *HSFA2*, *HSFA6B*, *HSFA6A*, *HSFA4A*, *HSFB2B*, *HSFB3*, *HSF4*, *NAC046*, *NAC032*, *NAC087*, *ZF2*, *CZF1*, *ZF2* were directly affected by this TF (Fig. 7, supplementary 6). This is the first report that showed this protein is a hub TF in response to salt and drought stresses.

*DREB2A* was affected by *RHL41*, *HSFB2A*, *NAC06* and *NAC096* whereas 15 genes such as *NAC019*, *RD26*, *HSFA4A*, *HSFA3*, *HSFA2*, *AT-HSFB2B* and etc. were affected by this TF (Fig. 7, supplementary 6). DREB (dehydration responsive element binding) is a subfamily of AP2/ERF transcription factors family that regulate ABA-independent signal transduction pathway [61]. *DREB2A* has one AP2/ERF domain and includes DRE-binding proteins (DREBs) which binds to dehydration-responsive element/C-repeat (DRE/CRT) in promoter of abiotic stress-responsive genes and regulates their expression [32, 37]. It has been previously shown that the expression of *DREB2A* was induced in response to dehydration, salt and heat stresses (5, 32, 37). Based on the gene network analysis, *HSFB2A*, *RHL41*, *DREB2A*, *ERF104* and *HY5* genes affected *ZAT6* expression (Fig. 7, supplementary 6). This TF activated the expression of some genes encoding TFs such as *RD26*, *HSFA4A*, *ERF-1*, *WRKY25* and *NAC047* (Fig. 7). *ZAT6* belongs to C2H2 subfamily of Zinc-finger transcription factors family [62]. Based on the results of Liu *et al* (2013), salt and osmotic stresses regulated *ZAT6* expression in *Arabidopsis*. The expression of *RHL41* directly activated by *ERF104* and *HSFB2A* genes, and affected 14 genes such as *DREB2A*, *HSFA2*, *HSFA4A*, *WRKY25*, *ZAT6 ERF-1* and etc (Fig. 7, supplementary 6).

*RHL41* (AT5G59820) which also known as *ZAT12* is a member of C2H2 zinc finger transcription factors family [63]. The expression of *ZAT12* induced by light, low and high temperature, wounding, osmotic, salt and oxidative stresses [64, 65]. It has been also proved *ZAT12* involved in the up-regulation of *WRKY25* in response to oxidative stress, which is one of the genes in ROS signal transduction [66]. Based on our knowledge, there are no reports to indicate that, *AT-HSFB2B* and *HSFA4A* play important roles in salt and drought stresses, and this is the first report to show these members of HSF family may play as hub proteins in these stresses.

Network analysis interestingly showed that *STZ* (salt tolerance zinc finger), a TF from C2H2 family, is affected by 17 other TFs which is the highest number in this network, but has no effect on other TFs (outdegree equal zero) (Fig. 7, supplementary 6). It has been previously shown that this protein acts as a transcription repressor to elevate abiotic stress tolerance in plants [67]. Hence, it can be resulted that *STZ* has a critical role in stress tolerance mechanism in *Arabidopsis* ​and can be considered as a suitable candidate to enhance salt and drought tolerance in crop plants through genetic engineering approach.

## 4 Conclusion

Drought and salt stresses are two main groups of abiotic stresses which affect agriculture productivity in worldwide. Furthermore, there is a crosstalk between abiotic and biotic stresses in plants. Therefore, identification of molecular mechanisms in response to them is helpful to increase simultaneously tolerance to drought and salt stresses. To achieve deeper understanding of molecular mechanisms in *Arabidopsis* ​and identification of responsive genes to drought and salt stresses, we identified up-regulated DEGs in leaf and root tissues in response to these stresses using microarray data analysis approach. We found that there are some differences between roots and leaves in terms of DEGs. Generally, the number of DEGs which up-regulated in response to stresses in roots was 1.5-fold higher than leaves, especially for drought stress, whereas it can be concluded that the number of common pathways between roots and leaves induced by salt stress is more than those of induced by drought stress. Various proteins involved in stress response mechanisms that transcription factors are most important of them; among these kind of proteins, three families such as HSF, AP2/ERF and C2H2, appear to play critical roles in salt and drought stress response mechanisms in *Arabidopsis*. In addition, the results of this study introducing some new proteins such as *STZ*, a member of AP2/ERF family, which plays a critical role in stress tolerance mechanisms in *Arabidopsis* ​and can be considered as a suitable candidate for increasing salt and drought tolerance in crop plants.

## Declarations

### Author contribution statement

Razieh Ghorbani: Performed the experiments; Analyzed and interpreted the data; Contributed reagents, materials, analysis tools or data; Wrote the paper.

Abbas Alemzadeh: Conceived and designed the experiments; Analyzed and interpreted the data.

Hooman Razi: Contributed reagents, materials, analysis tools or data.

### Funding statement

This work was supported by Shiraz University.

### Competing interest statement

The authors declare no conflict of interest.

### Additional information

No additional information is available for this paper.
